# m6A modification: a novel mechanism that regulates atherosclerosis via macrophage polarization

**DOI:** 10.3389/fimmu.2025.1607932

**Published:** 2025-06-16

**Authors:** Xiaying Li, Hengkai Zhang, Yan Zhou, Lei Zhang, Ye Huang

**Affiliations:** ^1^ Graduate School, Beijing University of Chinese Medicine, Beijing, China; ^2^ Emergency Department, Xiyuan Hospital, China Academy of Chinese Medical Sciences, Beijing, China; ^3^ Emergency Department, Shanxi Traditional Chinese Medical Hospital, Shanxi, China

**Keywords:** macrophage polarization, cytokines, atherosclerosis, N 6 -methyladenosine modification, inflammatory

## Abstract

Atherosclerosis is a chronic vascular inflammatory disease in which macrophages play a pivotal role in modulating its pathology. In response to the intraplaque microenvironment, both pro-inflammatory M1 and anti-inflammatory M2 phenotypes of macrophages have the polarization capability, each influencing the inflammatory state through the secretion of distinct cytokines. N6-methyladenosine (m6A) modification, the most prevalent internal chemical modification of RNA, significantly impacts various biological processes, including RNA transcription and protein expression. m6A modification acts as a critical determinant in macrophage polarization, with its molecular mechanisms intricately linked to the progression of atherosclerosis. This review aims to elucidate how different macrophage polarization phenotypes influence the progression of atherosclerosis while also exploring the significance of m6A modifications in this pathological context, thereby providing a theoretical foundation for identifying novel diagnostic and therapeutic targets for atherosclerosis.

## 1 Introduction

Cardiovascular and cerebrovascular diseases represent a major category of disorders that pose a substantial threat to human health and life. Globally, approximately 16.7 million individuals die each year from cardiovascular diseases, making them the leading cause of mortality worldwide. Atherosclerosis (AS), recognized as the primary etiological factor for myocardial necrosis, ischemic stroke, coronary artery disease, and other cardiovascular condition (1), is characterized by arterial wall thickening and stiffening, elasticity loss, lumen narrowing, and yellow atheromatous plaques accumulation in the intima, qualifying as a chronic vascular inflammatory disease with complex pathophysiology. Theories including lipid infiltration, oxidation, and injury response have been proposed to elucidate its pathogenesis. Currently, it is widely accepted that endothelial cells, smooth muscle cells, macrophages, and lymphocytes play crucial roles in the development of AS (2). In the pathogenesis of AS, macrophages play a pivotal role in driving AS progression. They prompt to plaque formation, degrade components of the fibrous cap and necrotic core, intensify inflammatory responses, and induce apoptosis of smooth muscle cells and leukocytes within the plaque (3). Additionally, macrophages are actively involved in lipid metabolism by regulating proliferation and migration, secreting various inflammatory factors, and phagocytosing lipids, dead cells, and other debris (4, 5). Shaped by a complex interplay of microbial factors and cytokine networks in their immediate microenvironment, macrophages, as one of the most plastic cell types in the immune system, exhibit diverse polarization states upon activation (6). These polarization states result in the classification of macrophages into various subtypes based on their functional characteristics and surface molecule expression. Specifically, M1 and Mox macrophages promote lesion development through the expression of pro-inflammatory factors (7), while M2 and Mhem macrophages mitigate disease progression by exerting anti-inflammatory effects (8). The pivotal role of macrophages in AS is closely tied to these diverse polarization subtypes, which are integral to all aspect of AS pathogenesis (9). Epigenetic modifications serve as regulatory mechanisms that influence gene expression without altering the fundamental DNA sequence, thereby shaping various biological processes such as gene expression, aging, and disease development (10). Among these modifications, N^6^-methyladenosine (m6A) is one of the most prevalent and abundant epigenetic marks found in eukaryotic RNAs (11). Studies have demonstrated that m6A modification plays a crucial role in tissue and cellular metabolic processes associated with AS (12, 13). Specifically, m6A modification has been shown to promote endothelial inflammatory responses and facilitate the formation of AS plaque, whereas inhibiting m6A modification reduces endothelial inflammation and mitigates AS progression in mice (14). The established roles of m6A modification in regulating tissue cell metabolism and promoting atherosclerotic plaque formation highlight its potential to elucidate macrophage polarization as a critical frontier in atherosclerosis mechanism research. In this review, we investigate the association between distinct macrophage polarization subtypes and AS, as well as the impact of m6A modification on macrophage polarization in AS. This review aims to provide novel insights and solid evidence for the clinical diagnosis and treatment of AS.

## 2 Role of macrophage polarization in AS

### 2.1 Macrophage polarization

Macrophages undergo polarization into distinct subtypes in response to stimuli from the intraplaque microenvironment. These subtypes display characteristic gene and protein expression profiles (15, 16). Specifically, macrophages can polarize into two primary subpopulations: classically activated macrophages (M1) and alternatively activated macrophages (M2), each exhibiting distinct functional properties (17).

M1 macrophages originate from Ly6C^high^ monocytes, establishing pro-inflammatory traits and marked by CD86 and CD16/32 surface markers (18). These cells are primarily triggered by TLR ligands such as lipopolysaccharide (LPS) and Th1-type cytokines like interferon-γ. LPS activates the canonical NF-κB pathway, leading to increased expression of inflammatory genes including TNF-α and interleukin-12 (IL-12), both of which are hallmarks of M1 polarization. Interferon-γ, on the other hand, activates signal transducer and activator of transcription 1 (STAT1), promoting the expression of downstream target genes in M1 macrophages, such as inducible nitric oxide synthase (iNOS) and IL-12 (19). Suppressor of cytokine signaling-3 (SOCS3), an inhibitor of the JAK/STAT signaling pathway, is induced via interferon signaling and plays a role in suppressing the M1 pro-inflammatory phenotype (20). Actually, M1 macrophages exhibit potent antimicrobial and antitumor activities, while also contributing to ROS-induced tissue damage (21).

M2 macrophages originate from Ly6C^low^ monocytes, which exhibit anti-inflammatory properties (18). These monocytes are polarized into the M2 phenotype by Th2-type cytokines IL-4 or IL-13 through the activation of STAT6 via IL-4Rα (22). Additionally, these macrophages express mannose receptor, arginase 1, and dectin-1 on their surface (23, 24). The IL-4/STAT6 pathway induces ligand-dependent peroxisome proliferator-activated receptor-γ (PPAR-γ) expression, thereby facilitating M2 polarization (25). PPAR-γ upregulates various subsets of genes related to anti-inflammatory function, including arginase 1, cysteine-rich secretory proteins, and IL-10 (26), all of which are crucial for maintaining the M2 phenotype. M2 macrophages act as a pivotal factor in immune regulation, tissue remodeling, wound healing, and angiogenesis (27, 28).

### 2.2 Effects of different polarization states on AS

M1 and M2 macrophages exhibit distinct functions and are distributed in different regions within the atherosclerotic plaque. These differences influence the development of AS due to their varying protein expression patterns and levels. The progression of AS is characterized by a reduction in number of M2 macrophages within the plaque, a decreased M2/M1 ratio, and elevated levels of inflammatory factors and matrix metalloproteinases (29, 30). Furthermore, the diverse polarization phenotypes of macrophages can affect plaque regression by promoting plaque size reduction and enhancing stability (31, 32).

Atherosclerotic plaques can be categorized into stable and unstable types. Unstable plaques, also known as vulnerable plaques, are characterized by their rapid progression and higher risk of rupture and thrombus formation. M1 macrophages predominate in unstable plaques and secrete elevated levels of pro-inflammatory cytokines, inducible iNOS, and ROS (8). After lipid uptake, M1 macrophages produce and secrete high level of pro-inflammatory cytokines, including IL-1, IL-6, IL-12, IL-15, IL-18, and TNF-α, through multiple complex mechanisms, thereby triggering a robust inflammatory response. The expression of TNF-α is regulated by the p38 mitogen-activated protein kinase (p38MAPK)/NF-κB pathway (33), which upregulates vascular cell adhesion molecule-1 (VCAM-1) expression and promotes atherogenesis (34, 35). In contrast, IL-6 expression is controlled by the STAT pathway (36) and influences nearly all cells involved in atherogenesis by enhancing cytokine and adhesion molecule expression, as well as promoting the migration and proliferation of vascular smooth muscle and endothelial cells (37).

M2 macrophages play a crucial role in plaque stabilization, comprising a substantial proportion of the plaque microenvironment. They secrete cytokines such as transforming growth factor-β (TGF-β) and IL-10 (38). TGF-β not only inhibits natural killer cell activity and TNF-α production but also stimulates collagen synthesis and fibroblast proliferation, thereby reinforcing the fibrous cap and enhancing plaque stability (39) IL-10 reduces TNF-α production, downregulates the expression of intercellular adhesion molecule-1 (ICAM-1) on endothelial cells, facilitates the polarization of M1-type macrophages towards the M2 phenotype, and exhibits anti-AS effects (**Table 1**) (40, 41).

**Table 1:** Functions of M1/M2-type macrophages and their relationship with AS.

Subtype	Role	Distribution	Secreted factors	Regulatory pathways	Relationship with AS
M1 macrophages	Pro-inflammatory	Plaque shoulders susceptible to rupture	TNF-α and IL-6 iNOS and ROS	p38MAPK/NF-κB STAT	Upregulate VCAM-1 expression and promote AS Increase the contents of pro-inflammatory factors and fibrinogen (42)
M2 macrophages	Anti-inflammatory	Stabilized plaque regions/tunica adventitia	TGF-β and IL-10	PPAR-γ	Reduce TNF-α expression, protect the fibrous cap, and stabilize plaque Reduce ICAM-1 expression and promote conversion from M1-type to M2-type

The shoulder region of atherosclerotic plaques, being the thinnest part, is particularly susceptible to rupture (43) M1 macrophage markers are predominantly localized in the shoulders of rupture-prone plaques, while M2 macrophage markers are primarily observed in the tunica adventitia or stable plaque regions (44). Moreover, cytokines derived from both M1 and M2 macrophage induce osteogenic differentiation and mineralization of smooth muscles cells, thereby promoting arterial vascular calcification, which subsequently affects the stability of AS plaques (45). Calcification, a hallmark of AS, is considered one of the principal risk factors for plaque rupture (46). Both M1 and M2 macrophages play significant roles in vascular calcification. The initial calcium deposition in the necrotic core of the lesion by M1 macrophages is termed microcalcification (47), whereas calcium deposition associated with M2 macrophages is referred to as macrocalcification. Macrocalcification tends to stabilize plaque, while microcalcification increases the likelihood of plaque rupture (48).

Importantly, M1 and M2 macrophages exhibit plasticity and can interconvert. Feig et al. (49) utilized a transgenic approach to enhance lipid metabolism in mice, demonstrating that M2 macrophages are reprogrammed into M1 macrophages during plaque progression. Conversely, during atherosclerotic plaque regression, the expression of CD68^+^ inflammatory cells decreases within the plaque, while the expression of M2 macrophage marker genes increases, indicating a shift from M1-type to M2-type macrophages. These findings suggest that the balance between M2 to M1 macrophages may play a critical role in both the progression and regression of AS plaques.

Harmon et al. (50) induced the transformation of intraplaque macrophages toward the M2 macrophages in ApoE^-/-^ mice and observed reduced intraplaque inflammatory factor activity and enhanced plaque stability, as evidenced by histological analysis. This indicates that M2 macrophages play a crucial role in mitigating the inflammatory response and stabilizing atherosclerotic plaques in hypercholesterolemic mice. Rahman et al. (51) compared M2 macrophages expression between chemokine receptor-deficient mice and normolipidemic controls, revealing significant plaque regression and increased M2 macrophage labeling in that chemokine receptor-deficient mice. These findings indicate that M2 macrophages are essential for reducing inflammation and promoting plaque regression.

However, M2 macrophages have been demonstrated to facilitate foam cell formation and augment the number of foam cells in atherosclerotic lesions (52), suggesting that their role in AS progression, particularly concerning foam cell formation, is still under debate. This paradoxical phenomenon can be attributed to the heterogeneity of M2 macrophages or disparities in the microenvironment. M2 macrophages can be further categorized into M2a, M2b, and M2c subtypes, each potentially exhibiting distinct functions in lipid metabolism and inflammatory modulation (53). Moreover, microenvironmental signals—such as plaque lipid composition, cytokine hyperactivation, or hypoxic stress—may steer their differentiation pathways (54), thereby enabling M2 macrophages to exhibit dual roles in both “promoting foam cell formation” and “facilitating plaque stabilization”. As a result, the role of M2 macrophages in atherosclerosis (AS) is not unidirectionally protective. Unraveling the mechanistic interplay between M2 macrophages and foam cell formation required more in-depth investigations, particularly focusing on the functional profiling of different M2 subtypes various pathological stages.

## 3 m6A modification and macrophage polarization

### 3.1 General molecular mechanisms processes of m6A modification

m6A is a reversible biological modification characterized by the methylation of the amino group at position 6 of adenosine. This modification predominantly localizes to the termination codon, the 3’untranslated region (UTR), and the exon RRACH sequence (55). m6A exhibits conservative, dynamic and reversible properties, which are regulated by various proteins and enzymes, including methyltransferase, demethylase, and m6A-specific binding proteins (56). This epigenetic modification influences multiple cellular processes such as RNA splicing, processing, mRNA stability, and gene expression regulation, thereby playing a crucial role in mammalian development and disease progression (57).

In the nucleus, the methyltransferase complex and demethylases collaboratively regulate the distribution and extent of m6A modification on RNA. Meanwhile, reader proteins control the selective splicing, nuclear export, translation, and degradation of m6A-modified RNA (58).

#### 3.1.1 m6A methyltransferases

The methyltransferase complex, often referred to as the “writer”, plays a crucial role in catalyzing the transfer of methyl groups. This complex primarily comprises methyltransferase-like (METTL) enzymes such as METTL3, METTL14, and METTL16, along with the regulatory subunit Wilms’ tumor 1-associating protein (WTAP) (59). METTL3 is the catalytically active subunit that utilizes S-adenosylmethionine to provide the methyl group, thereby playing a pivotal role in the methylation process (60). As the core enzyme of the methyltransferase complex, METTL3 not only catalyzes methyl transfer but also influences gene expression by stably recruiting the complex to specific gene loci and facilitating RNA transport to ribosomes (61). METTL14 provides an RNA-binging platform, that synergistically enhances the methytransferase’s affinity for RNA substrates, thereby stabilizing the complex (62). Although WTAP lacks methyltransferase activity, it interacts with the METTL3–METTL14 complex and plays a critical role in modulating the nuclear localization of the complex, thus influencing the efficiency of methylation (63, 64).

#### 3.1.2 m6A demethylases

The primary function of m6A demethylases, also known as erasers, is to remove methyl groups from RNAs bearing m6A modification. These demethylases include the fat mass and obesity-associated (FTO) and the alkylated DNA repair enzyme ALKB homolog 5 (ALKBH5) (65). Both proteins belong to the ALKB family but employ distinct mechanisms for demethylation. Specifically, ALKBH5 catalyzes demethylation in a single step without generating intermediate products (66), while FTO primarily catalyzes demethylation via an oxidation reaction that requires Fe (II) and 2-oxoglutarate (67). This process yields two intermediates, N^6^-hydroxymethyladenosine and N^6^-formyladenosine, which ultimately transform into adenine and formaldehyde (71). Moreover, FTO can demethylate various nucleotides, including m6A, whereas ALKBH5 specifically targets m6A (68).

#### 3.1.3 m6A-binding proteins

m6A methylation sites are recognized by a variety of m6A-binding proteins, which play essential roles in regulating RNA metabolism, processing, translation, and stability. These m6A-binding proteins(readers), specifically recognize m6A modifications and predominantly comprise YTH domain-containing proteins, including the YTHDF family (YTHDF1, YTHDF2, and YTHDF3) and YTHDC family (YTHDC1 and YTHDC2) (69). These proteins bind to m6A modification sites on RNA molecules, regulating translation efficiency and stability of m6A-modified mRNAs to influence various physiological and pathological processes (70).

YTHDF2 was identified as the first m6A-binding protein that promotes mRNA degradation and destabilizes target transcripts (71). YTHDF1 enhances mRNA translation, thereby promoting protein synthesis. YTHDF3, which is closely related to YTHDF1 and YTHDF2, plays a crucial role in facilitating the specific binding of YTHDF1and YTHDF2 to RNA. Functionally, YTHDF3 collaborates with YTHDF1 to boost the translation of methylated mRNA, facilitate protein synthesis, and augment YTHDF2-mediated mRNA degradation and instability (72). By integrating and cooperating with m6A methylation modifications, YTHDF1–3 collectively regulate mRNA metabolism, processing, translation, and degradation. In contrast, YTHDC1 regulates nuclear mRNA splicing (73) and mediates nuclear export via RNA methylation (74). YTHDC2, featuring multiple RNA-binding domains, is a key m6A-binding protein in reproductive system development and maturation. It enhances mRNA translation efficiency by binding to m6A sites (75).

**Figure 1:**
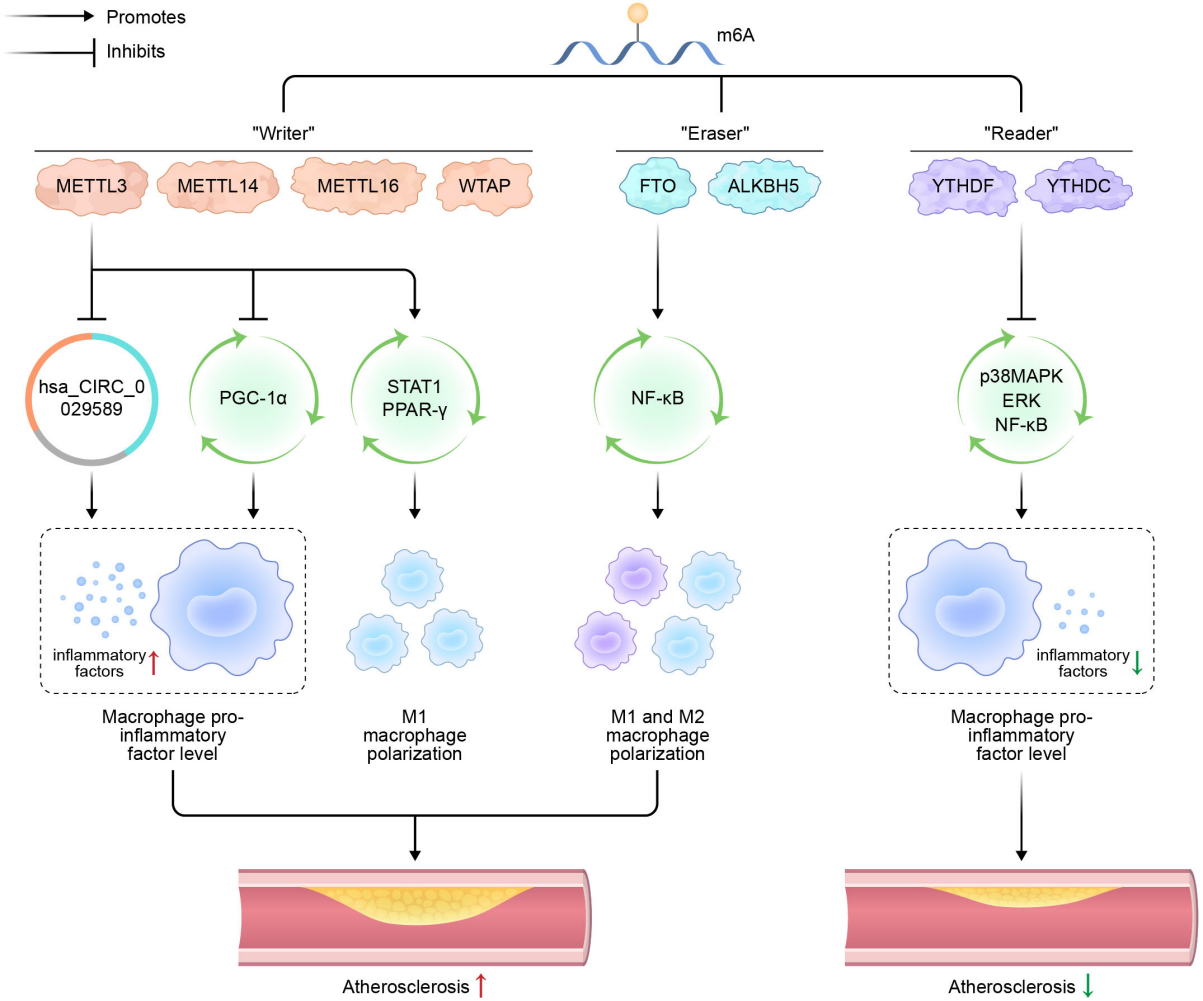
Schematic representation of m6A modification, macrophage polarization, and atherosclerosis.

m6A modification significantly multiple facets of mRNA metabolism, including stability, translation efficiency, alternative splicing, and intracellular transport. This epigenetic mark serves an indispensable function in a broad spectrum of physiological and pathological processes, such as stem cell differentiation, immune response modulation, and tumorigenesis (**Figure 1**) (76, 77).

N^6^-methyladenosine (m6A); methyltransferases-like (METTL); Wilms’ tumor 1-associating protein (WTAP); fat mass and obesity-associated (FTO) protein; alkane hydroxylase homolog 5 (ALKBH5); circular RNA (hsa_CIRC_0029589); peroxisome proliferator-activated receptor-γ coactivator (PGC)-1α; signal transducer and activator of transcription 1 (STAT1); peroxisome proliferators-activated receptors (PPARs); nuclear factor kappa-B (NF-κB); p38 mitogen-activated protein kinase (p38MAPK).

### 3.2 Effect of m6A modification on AS

Cellular structural damage and dysfunction caused by endothelial cell inflammation are pivotal mechanisms in the pathogenesis and progression of AS. D. Jian et al. (14) demonstrated through RNA co-immunoprecipitation experiments that METTL14 directly interacts with FOXO1 mRNA and recognizes its methylation sites via YTHDF1, thereby enhancing the translation efficiency of FOXO1 mRNA. The knockout of METTL14 reduces inflammation-induced FOXO1 expression and alleviates endothelial cells inflammation, consequently slowing the progression of AS. These findings suggest that m6A methylation plays a crucial role in vascular endothelial cell inflammation and the development of AS.

Moreover, it was observed that Atorvastatin reduced the expression of FTO in vascular endothelial cells. Knockdown of FTO led to upregulation of Kruppel-like factor 2 (KLF2) and endothelial nitric oxide synthase (eNOS), while simultaneously downregulating endothelial expression of VCAM-1 and ICAM-1, which are typically induced by inflammatory stimuli. By employing methylated RNA co-immunoprecipitation coupled with dual-luciferase reporter assays, researchers demonstrated that FTO directly interacts with KLF2 and eNOS. This interaction enables the modulation of their expression via an m6A modification-dependent mechanism (78).

X. Zhang et al. (79) demonstrated that ALKBH5 expression is downregulated in endothelial cells undergoing TNF-α-induced apoptosis. Upon upregulation of ALKBH5, the apoptotic rate of TNF-α-treated endothelial cells decreased, accompanied by an increase in BCL2 expression. Importantly, silencing BCL2 abolished the protective effect of ALKBH5 on TNF-α-induced apoptosis, indicating that ALKBH5 exerts its anti-apoptotic function in vascular endothelial cells through upregulation of BCL2.

Furthermore, it has been proved that m6A methylation is of crucial importance in regulating the angiogenesis of endothelial cells. Specifically, METTL3 knockdown continuously activates the Notch signal pathway, thereby significantly influencing endothelial cell angiogenesis (80). WTAP, another critical subunit of the methyltransferase complex, regulates desmosome protein expression through m6A methylation. Decreased WTAP expression can inhibit endothelial cell angiogenesis (81). Additionally, ALKBH5 has been shown to maintain endothelial cell angiogenesis under acute ischemic stress by reducing SPHK1 m6A methylation and modulating downstream eNOS-AKT signal transduction (82).

Vascular smooth muscle cells (VSMCs) exhibit phenotypic and functional plasticity in response to vascular injury. Following vascular injury, VSMCs can transition from a quiescent, contractile phenotype to a synthetic state, which is marked by elevated rates of proliferation, migration, secretion and production of extracellular matrix components. Chronic inflammatory stimulation impairs the regulatory functions of VSMCs, leading to reduce contractile capacity and heightened secretory activity. This ultimately results in arterial wall thickening and stenosis (2).

The occurrence of AS is closely associated with post-angiogenesis restenosis. Intimal hyperplasia, primarily driven by the proliferation and migration of VSMCs, is one of the key factors contributing to arterial restenosis. Zhu et al. (83) investigated m6A methylation in the carotid arteries of rats following balloon injury. Their study demonstrated that both m6A methylation levels and WTAP expression were significantly reduced after injury. However, it was found that administration of total saponins extracted from *Panax notoginseng* increased WTAP expression, thereby enhancing the m6A methylation of the downstream target gene p16. This process concurrently suppressed the proliferation and migration of VSMCs. These findings suggest that WTAP regulates p16 expression via m6A methylation, consequently modulating the viability, proliferation, and migration of VSMCs.

Similarly, B.-F. Zhang et al. (84) demonstrated that m6A methylation plays a critical role in the insulin resistance-induced abnormal proliferation of VSMCs. Their study revealed elevated FTO levels in VSMCs treated with insulin and in type 2 diabetic mice with intimal injury. Furthermore, genetic ablation of FTO significantly suppressed the insulin-induced pathological proliferation and migration of VSMCs.

Y. Qin et al. (85)demonstrated that under hypoxic conditions resulted in elevated expression of METTL3 and YTHDF2 in pulmonary arterial smooth muscle cells (PASMCs) as well as in a rodent model subjected to hypoxia. Silencing METTL3 was found to significantly reduce the proliferation and migratory capabilities of PASMCs. In another study, it was reported that hypoxia-induced adipose-derived stem cells (ASCs) exhibited upregulated expression of METTL3, along with increased secretion of paracrine factors such as vascular endothelial growth factor (VEGF) and transforming growth factor-beta (TGF-β), which promoted their differentiation into VSMCs. These findings indicate that hypoxic stress not only promotes the differentiation of ASCs into VSMCs but also coordinates the expression of paracrine factors via METTL3 regulation consequently affecting cell migration, proliferation, and differentiation processes (86).

To sum up, existing studies have demonstrated that m6A methylation not only influences the biological functions of vascular endothelial cells but also modulates the activity, migration, and proliferation of VSMCs, thereby affecting the pathogenesis and progression of AS.

### 3.3 Effect of m6A on macrophage polarization

The role of m6A in the immune system is evident across three critical areas: immune recognition, activation of both innate and acquired immune responses, and differentiation immune cells. For example, specific deletion of METTL3 in CD4^+^ T cells disrupts their dynamic homeostasis and differentiation (87). Furthermore, viral infection in host cells leads to reduced expression of the m6A eraser ALKBH5, which subsequently modulates cellular metabolism to inhibit viral replication (88).

Regarding macrophage polarization, Liu et al. (89) induced M1 polarization in mouse peritoneal macrophages and observed a significant upregulation of METTL3 at both mRNA and protein levels, suggesting a robust correlation between METTL3 expression and M1 polarization in these cells. In subsequent experiments, METTL3 was either inhibited or overexpressed in mouse bone marrow-derived macrophages (BMDMs) using transfection techniques. The expression levels of key regulators of macrophage polarization, including interferon regulatory factor-5 (IRF5), STAT1, and NF-κB, were subsequently evaluated. The findings demonstrated that METTL3 mediates *STAT1* mRNA methylation, thereby enhancing its mRNA stability, upregulating STAT1 expression, promoting M1 macrophage polarization, inhibiting M2 macrophage polarization, and participating in inflammatory responses.

Furthermore, a significant relationship has been established between the m6A demethylase FTO and macrophage polarization. Gu et al. (6) demonstrated that FTO expression was significantly downregulated during both M1 and M2 polarization of BMDMs, where M1 polarization was induced by *E. coli* LPS and interferon-γ, while M2 polarization was triggered by IL-4. Notably, the suppression of FTO expression impaired the polarization of both M1 and M2 macrophage. Further investigations revealed that FTO modulates the NF-κB signaling pathway and influences the stability of STAT1 and PPAR-γ. Specifically, FTO knockdown inhibits the NF-κB signaling cascade and destabilizes STAT1 and PPAR-γ, which are essential for macrophage polarization mediated by YTHDF2. These findings underscore the critical role of FTO in regulating macrophage polarization.

hsa_CIRC_0029589, a circular RNA, is upregulated in vascular smooth muscle cells of hyperlipidemia patients (90). Guo et al. (91) reported that in peripheral blood macrophages from patients with acute coronary syndrome, hsa_CIRC_0029589 expression is decreased while METTL3 expression is increased. Inhibition of METTL3 leads to a decrease in the methylation level of hsa_CIRC_0029589, resulting in its upregulation. Conversely, interferon regulatory factor-1 (IRF-1) enhances METTL3 expression within macrophages, thereby suppressing hsa_CIRC_0029589 expression. These findings suggest that IRF-1 inhibits the expression of hsa_CIRC_0029589 via enhanced m6A modification, thereby subsequently facilitating macrophage pyroptosis and amplifying inflammatory responses in AS.

Cai et al. (92) recently demonstrated that in endotoxin-stimulated macrophages, the knockout of METTL3 leads to a significant reduction in its expression, along with an upregulation of pro-inflammatory cytokines such as TNF-α and IL-6. Further experiments demonstrated that METTL3 deletion inhibits YTHDF2-mediated degradation of NOD1 and RIPK2 mRNA, resulting in upregulation of the NOD1 pathway and subsequent enhancement of lipopolysaccharide-induced macrophage inflammation. These findings suggest that METTL3 negatively regulates LPS-induced inflammation.

Additionally, Yu et al. (93) shown that inhibiting YTHDF2 in LPS-stimulated RAW264.7 macrophages enhances the stability of MAP2K4 and MAP4K4 mRNA. This stabilization subsequently enhances the activation of inflammatory pathways for instance p38MAPK, extracellular signal-regulated kinase (ERK), and the NF-κB pathway, resulting elevated levels of downstream inflammatory molecules, including IL-6, TNF-α, IL-1β, and IL-12. These findings indicate that YTHDF2 acts as a negative regulator of the LPS-induced inflammatory response in macrophages. Furthermore, X. Zhang et al. (94) Showed that METTL3 and YTHDF2 jointly suppress PGC-1α expression in THP-1 macrophages, leading to increased accumulation of cellular and mitochondrial ROS, and heightened expression of pro-inflammatory cytokines.

## 4 Summary and outlook

Macrophages exhibit significant heterogeneity in AS. As key components of the innate immune system, macrophages play a crucial role in regulating various physiological and pathological states through their M1 and M2 polarization, which substantially influences AS progression. Specifically, M1-type macrophages secrete pro-inflammatory factors, whereas M2-type macrophages produce anti-inflammatory factors. The dynamic shifts in the quantity and proportion of these two phenotypes within plaque tissue have a substantial impact on plaque development. m6A, a reversible RNA modification, plays a critical role in disease and treatment by modulating gene expression. Moreover, m6A profoundly affects macrophage polarization and cytokine secretion in AS. A thorough investigation into the interactions among polarization, m6A modification, and AS development is essential for identify novel targets for diagnosing and treating AS.
